# DoubleSG-DTA: Deep Learning for Drug Discovery: Case Study on the Non-Small Cell Lung Cancer with *EGFR*^*T*790*M*^ Mutation

**DOI:** 10.3390/pharmaceutics15020675

**Published:** 2023-02-16

**Authors:** Yongtao Qian, Wanxing Ni, Xingxing Xianyu, Liang Tao, Qin Wang

**Affiliations:** Department of Pharmacology, Zhongshan School of Medicine, Sun Yat-sen University, Guangzhou 510080, China

**Keywords:** drug–target affinity, graph isomorphism network, squeeze-and-excitation network, cross-multi-head attention, drug discovery, non-small cell lung cancer

## Abstract

Drug–targeted therapies are promising approaches to treating tumors, and research on receptor–ligand interactions for discovering high-affinity targeted drugs has been accelerating drug development. This study presents a mechanism-driven deep learning-based computational model to learn double drug sequences, protein sequences, and drug graphs to project drug–target affinities (DTAs), which was termed the DoubleSG-DTA. We deployed lightweight graph isomorphism networks to aggregate drug graph representations and discriminate between molecular structures, and stacked multilayer squeeze-and-excitation networks to selectively enhance spatial features of drug and protein sequences. What is more, cross-multi-head attentions were constructed to further model the non-covalent molecular docking behavior. The multiple cross-validation experimental evaluations on various datasets indicated that DoubleSG-DTA consistently outperformed all previously reported works. To showcase the value of DoubleSG-DTA, we applied it to generate promising hit compounds of Non-Small Cell Lung Cancer harboring $EGFR^{T790M}$ mutation from natural products, which were consistent with reported laboratory studies. Afterward, we further investigated the interpretability of the graph-based “black box” model and highlighted the active structures that contributed the most. DoubleSG-DTA thus provides a powerful and interpretable framework that extrapolates for potential chemicals to modulate the systemic response to disease.

## 1. Introduction

Clinically acquired resistance is an insurmountable dilemma for small-molecule kinase inhibitors to treat cancer [1]. Nevertheless, locating small-molecule ligands with high affinity and good properties for target proteins in a broad chemical space has been a primary challenge in drug research and development (R&D) [2]. To date, it cannot be overstated to describe the kinase drugs approved by The U.S. Food and Drug Administration (FDA) to overcome clinical resistance driven by protein kinase “gatekeeper” mutation as “desert oasis”. Lung cancer is the leading cause of cancer-related deaths worldwide, with non-small cell lung cancer (NSCLC) being the most common type of lung cancer. Secondary epidermal growth factor receptor (EGFR) mutations in threonine 790 (T790M) lead to acquired resistance which severely affects patient prognosis. Therefore, strategies or drugs to overcome resistance are urgent to prolong the survival of patients with NSCLC.

Laborious wet labs and high-throughput screening techniques are so time-consuming and challenging that they are unsuitable for screening candidate drugs from a broad range of compound groups in pre-drug R&D. With improvements in machine learning theory and an abundance of pharmacological data available, machine learning provides sufficient power for the development of precision medicine and artificially intelligent drug design (AIDD). Many encouraging scientific achievements have convincingly demonstrated the potential of these approaches. For instance, the knowledge graph (KG) enables to detect of the drivers of tumor resistance and adverse drug reactions in a wider multi-omics space [3,4]; reinforcement learning (RL) has been found to be particularly effective in the de novo design and multi-objective optimization of drug molecules [5,6,7]. Deep learning is a powerful data-driven algorithm in machine learning, which offers significant advantages to reveal implicit relationships between drugs, diseases, and genes that are not easily detected, owing to the powerful generalization and representation extraction capability. Some in silico methods that explore potential drug–target associations to advance drug R&D have been developed to narrow the research concentration areas toward the more workable drugs.

Some studies have viewed DTA prediction as a binary classification task, borrowing binary numbers (1/0) to label whether the two are combined [8,9,10], while some others treat it as a regression task and use floating-point numbers to indicate DTAs [11,12,13].

The random forest (RF) algorithm broke the previous methods of relying on multi-parameter scoring functions to infer DTA [14], which has proven to be convincing for extrapolating drug–target relationships in larger chemical spaces. KronRLS [15] and SimBoost [12] were regression-based machine-learning approaches that evaluated similarities between drugs and proteins to determine DTA. Various excellent deep-learning works have been presented. DeepDTA [8] and Attention-DTA [16] leveraged the convolutional neural networks (CNNs) to obtain the hidden relationships of atomic and amino acid sequences. DeepCDA incorporated the long- to short-term memory network which aims to alleviate the phenomenon of gradient disappearance and gradient explosion [17]. MATT-DTI deployed relation-aware self-attention with position embedding to reinforce relative positional associations among atoms [13]. Transformer-based works have come to the fore in various natural language processing (NLP) tasks. DMIL-PPDTA utilized the transformer encoder to enrich word embeddings of drug and protein sequences, aiming to learn hidden associations from the raw data [18]. DeepAtom [19] extrapolated node-level interaction information relevant to binding from the voxelized protein–compound complex structures. Nevertheless, these models rely on known 3D drug–target complexes, and the computational burden of complex 3D convolutional networks to extract the features of massive complexes is expensive. GraphDTA [11] and MGraphDTA [20] represented compounds as topological graphs and evaluated several types of Graph Neural Network (GNN) variants, including Graph Convolutional Network (GCN) [21], Graph Isomorphism Network (GIN) [22], and the Graph Attention Network (GAT) [23], with the aim of replacing CNN and achieving excellent performance. Additionally, DGraphDTA encoded both drugs and proteins into the graphs for inferring DTA by GNN [24]. Among those graph-based methods, they not only effectively avoid the drawbacks of few complex samples and high computational cost, but compensate for the problem of inadequate SMILES (Simplified Molecular Input Line Entry System) [25] for drug representation, and the molecule graph is closer to the natural description of compounds.

Although these methods produce excellent prediction results, they are difficult to generalize to real-world problems. Firstly, the molecular similarity principle [26] states that molecules with similar structures usually show similar biological activities and physicochemical properties; conversely, there are significant differences. Therefore, the model must discriminate between molecular structures over a wide chemical space. Moreover, modeling underlying complicated mapping patterns between compounds and proteins simply concatenate, which deviates from the non-covalent interaction between the receptor and ligand. More importantly, these approaches have limited interpretability as a result of the “black-box” property of graph neural networks. Considering that the false-positive statistics generated by the binary classification task directly impair the robustness of the model, here, predicting DTA was regarded as a regression problem. We propose a three-channel DoubleSG-DTA theoretical framework based on GINs and multiple attention mechanisms to address the aforementioned problems, which significantly outshines other regression-based SOTA methods on various benchmark datasets. Afterward, we visualize the gradient of atomic contributions in graph representations and compare them with the molecular docking poses to further extend the interpretability of the graph-based model.

This paper presents the main contributions as follows:

- DoubleSG-DTA combined graph isomorphism networks and the squeeze-and-excitation networks to extract multimodal representations of drugs in parallel, aiming to enhance the model to discriminate between compound structures and selectively suppress redundant information to disturb model decisions.
- The design of cross-multi-head attention mechanisms to model the reality-based non-covalent molecular docking behavior of drug substructures and subsequences with target proteins, respectively;
- Application of the DoubleSG-DTA to screen promising hit compounds of the NSCLC harboring $EGFR^{T790M}$ mutation from natural products, which have been consistent with reported laboratory studies.

## 2. Double Sequence and Graph to Predict Drug–Target Affinity (DoubleSG-DTA)

**Figure 1:**
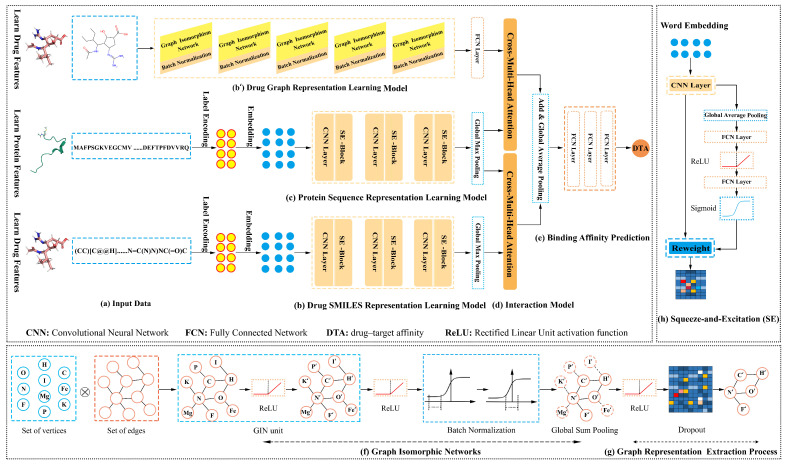
Architecture of the presented DoubleSG-DTA model.

This work developed the DoubleSG-DTA model with three-channel multimodal representations, four-channel interaction, and one-channel output for DTA prediction, which deployed multilayer GINs and multiple attention blocks, as shown in Figure 1. Primarily, we took the drug graphs and SMILES as inputs into the drug representation learning models. Multilayer GINs [22] and squeeze-and-excitation networks (SENets) [27] are jointly used as feature extractors for drugs. Additionally, the protein representation learning model captures the dominant feature of the over-redundant protein sequences that are highly dependent on stacked SENets. Moreover, to further encode the drug–target mutual interaction information, we designed cross-multi-head attention to model the reality-based non-covalent molecular docking behavior of drug substructures and subsequences with target proteins, respectively. Ultimately, we decoupled the attention coefficients into the Multilayer Perceptrons (MLPs) to predict DTA. This section presents the building blocks of our framework in order.

### 2.1. Word Embedding and Graph Encoding

Initially, we utilized high-dimensional word embeddings to uniquely encode drug and protein sequences. To this aim, we built label/integer dictionaries for drug SMILES and protein FASTA sequences, which consist of 64 and 22 key-value pairs, respectively. For example, the SMILES of Propylene glycol “CC(O)CO” and the $EGFR^{T790M}$ [28] protein subsequence “NWCVQIA” are encoded as [2222433312233] and [14212221581] according to the SMILES dictionary {‘C’:22, ‘N’:34, ‘O’:33, ‘(’:4, ‘)’:3} and the protein dictionary {‘A’:1, ‘N’:14, ‘C’:2, ‘Q’:15, ‘I’:8, ‘V’:22, ‘W’:21}. We then map each integer vector into word embeddings $D_{e}\in R^{l_{d}\times l_{e}}$ and $P_{e}\in R^{l_{p}\times l_{e}}$ by embedding layers. Where $l_{d}$ and $l_{p}$ denote the size of the SMILES and protein FASTA sequence, $l_{e}$ represents the embedding dimensions.

We convert SMILES to their corresponding molecular graphs $G=\left(V,E\right)$ and extract atom features by RDKit [29], where E and V are the sets of edges and atoms, respectively. Each atom node in a drug is represented by a multi-dimension vector of 10 molecular descriptors (atom symbol, atom number, hybridization, number of adjacent atoms, chirality, formal charge, aromaticity, number of bonded hydrogens, and explicit and implicit valence).

### 2.2. Drug and Protein Sequence Representation Learning Model

The CNNs construct text features by fusing spatial correlations between features that benefit from the convolutional kernel’s local receptive field but are likewise limited by it. In computer vision, the squeeze-and-excitation (SE) block with channel attention was integrated into existing architectures, which adaptively rescales channel-wise feature weights by explicitly modeling non-mutually-exclusive relationships between channels [27]. The research has confirmed that the SENets achieved superior performance for image classification with a slight increase in computational cost [27]. Accordingly, we stacked multilayer SENets designed to selectively enhance effective statistics and suppress noise to disturb model decisions. Given $U\in R^{H\times W\times C}$ as the feature matrix of the convolution layer output, we routed it to the SE block, where $U=[u_{1},u_{2},\cdots ,u_{C}]$.

SE module makes use of squeeze, excitation, and reweighting operators. The squeeze operator intrinsically aims to transform the dimensions of the feature matrix *U* and obtain channel-wise statistics $z\in R^{C}$ by applying the global average pooling operation.

(1) $$\begin{array}{c} z_{c}=F_{sq}\left(u_{c}\right)=\frac{1}{H\times W}\sum_{i=1}^{H}\sum_{j=1}^{W}u_{c}\left(i,j\right). \end{array}$$

The excitation module leverages two learnable FCNs with the gating mechanism to learn inter-channel non-linear interaction and filter non-dominant features.

(2) $$\begin{array}{c} s=F_{ex}\left(Z,W\right)=\sigma \left(W_{2}\delta \left(W_{1}z\right)\right), \end{array}$$

where the δ is the Rectified Linear Unit (ReLU) activation function, and σ is the sigmoid function, and $W_{1}\in R^{\frac{C}{r}\times C}$ and $W_{2}\in R^{C\times \frac{C}{r}}$ are the two learnable weight matrices. The reduction ratio was set to r = 16 to reconcile the balance between performance and complexity [27].

The reweighting representation $x_{c}$ was computed by applying the channel-wise multiplication operation to the channel attention weight $s_{c}$ and the feature map $u_{c}$.

(3) $$\begin{array}{c} x_{c}=F_{sc}\left(u_{c},s_{c}\right)=s_{c}\times u_{c}. \end{array}$$

where $X=\left[x_{1},x_{2},\ldots \ldots x_{C}\right]$, $x_{c}\in R^{H\times W}$.

The word embeddings $D_{e}$ and $P_{e}$ are directly fed into the convolutional layers, then delivered to the SE block accompanied by a global max pooling operation to calculate desired feature information. Hence, the drug and protein sequence representations can be expressed as:

(4) $$\begin{array}{c} D_{SENet}=gmp\left(SE\left(CNN\left(D_{e}\right)\right)\right)\\ P_{SENet}=gmp\left(SE\left(CNN\left(P_{e}\right)\right)\right). \end{array}$$

### 2.3. Drug Graph Representation Learning Model

Drug molecules are non-Euclidean chemical structures that consist of entities (atoms) and relations (bonds) with rich semantic information and complex spatial structures. This is essential for accurately discriminating between drug molecules and precisely predicting the binding affinity of different compound molecules with proteins. Nevertheless, that is beyond the reach of traditional GNNs.

Meanwhile, we take into account that drugs with similar substructures may react pharmacologically with target proteins with the same or similar protein binding pockets. Interestingly, graph isomorphism networks [22] with injectivity broadly follow a flexible message-passing scheme that enables atoms to recursively update semantic information through aggregating near and far neighboring atomic features. A sufficient number of iterations allows the GIN to be perfectly equipped with the most powerful ability to “read-out” drug graph representations and identify drug molecules.

GIN updates atom feature vectors via the MLPs, ensuring that GIN still satisfies injectivity after K-iterations of aggregation. The graph representation is obtained by summing all of the atom feature vectors in the drug. Formally, the kernel function of GINs updates atom feature vector $D_{v}^{k}$, and the drug graph representation $D_{GIN}$ is:

(5) $$\begin{array}{c} D_{v}^{k}={MLP}^{k}\left(\left(1+\varepsilon^{k}\right)\cdot D_{v}^{k-1}+\sum_{i\in N_{\left(v\right)}}D_{i}^{k-1}\right)\\ D_{GIN}=CONCAT\left(READOUT\left(\left\{D_{v}^{k}|v\in G\right\}\right)\right), \end{array}$$

where $N_{v}$ is a set of nodes adjacent to atom *i*. The READOUT function is a graph-level pooling function. We made ε a learnable parameter.

The successful construction of deep GINs is highly dependent on the ReLU activation function and batch normalization, while batch normalization can effectively alleviate the vanishing gradient and over-smoothing problems.

(6) $$\begin{array}{c} GIN^{\left(l+1\right)}\left(G\right)=BNLayer\left(GIN^{\left(l\right)}\left(G\right)\right)\\ D_{GIN}=Dropout\left(\delta \left(GIN^{\left(n\right)}\left(G\right),W\right)\right) \end{array}$$

where BNLayer denotes node-level batch normalization.

### 2.4. Drug Molecule and Target Protein Interaction Model

Drug molecules binding to target proteins is actually an identification relationship similar to the “lock and key” model. Inspired by previous attention-based methods [13,17,30], we constructed two cross-multi-head attention modules to model non-covalent molecular docking behavior between compounds and proteins, instead of simply connecting drug and protein representations that inherently generates more intrusive information. Concretely, we observed the associations among molecules’ substructures, subsequences, and residues from multiple independent perspectives. The cross-multi-head attention blocks take the drug and protein sequences feature matrices $D_{SENet}\in R^{l_{d}\times l_{c}}$ and $P_{SENet}\in R^{l_{p}\times l_{c}}$ of SENets, and the drug graph-level representation $D_{GIN}\in R^{l_{d}\times l_{g}}$ of the GIN as inputs, respectively.

In the following paragraphs, we construct learnable linear transition layers so that each head can fully learn from the high-dimensional features. Afterward, we combine $D_{SENet}$, $D_{GIN}$ with $P_{SENet}$ by adopting the cross-multi-head attention mechanism.

(7) $$\begin{array}{c} Q_{s}=\delta \left(D_{SENet}W_{senet}+b_{senet}\right),Q_{g}=\delta \left(D_{GIN}W_{gin}+b_{gin}\right)\\ K=\delta \left(P_{SENet}W_{senet}+b_{senet}\right),V=\delta \left(P_{SENet}W_{senet}+b_{senet}\right) \end{array}$$

where $W_{senet}\in R^{l_{c}\times l_{a}}$, $W_{gin}\in R^{l_{g}\times l_{a}}$, and $b_{senet}$, $b_{gin}$ are the learnable weights and bias terms, respectively. *Q*, *K*, and *V* represent queries, keys, and values vectors. An individual scaled dot–product attention module was expressed as mapping the *Q* with *K*-*V* pairs to the similarity matrix. Multi-head attention jointly concerned different representation subspaces at distinct positions by concatenating *h* individual attention units [31].

We obtained one of the cross-multi-head attention weight $A_{DP1}$ as follows:

(8) $$Attention\left(Q_{s},K,V\right)=Soft\max\left(\frac{Q_{s}K^{T}}{\sqrt{l_{c}/h}}\right)\cdot V$$

(9) $$\begin{array}{c} head_{i}=Attention\left(Q_{s}W_{i}^{Q},KW_{i}^{K},VW_{i}^{V}\right)\\ A_{DP1}=Concat\left[head{}_{1},\ldots ,head_{h}\right]W^{O} \end{array}$$

where $W_{i}^{Q}$, $W_{i}^{K}$, $W_{i}^{V}$, and $W^{O}$ are parameter matrices for learning linear projections. Next, another cross-multi-head attention coefficient $A_{DP2}$ was computed as:

(10) $$\begin{array}{c} Attention\left(Q_{g},K,V\right)=Soft\max\left(\frac{Q_{g}K^{T}}{\sqrt{l_{g}/h}}\right)\cdot V \end{array}$$

(11) $$\begin{array}{c} head_{j}=Attention\left(Q_{g}W_{j}^{Q},KW_{j}^{K},VW_{j}^{V}\right)\\ A_{DP2}=Concat\left[head{}_{1},\ldots ,head_{h}\right]W^{O} \end{array}$$

Afterward, we decoupled the attention weight $A_{DP}$ to obtain drug attention weight $\alpha_{d}$ and protein attention weight $\alpha_{p}$ by applying row-wise sum and column-wise sum operations. We updated the drug representation $\alpha_{D}$ and protein representation $\alpha_{P}$.

(12) $$\begin{array}{c} A_{DP}=Concat\left[A_{DP1},A_{DP2}\right] \end{array}$$

(13) $$\begin{array}{c} \alpha_{D}=C\mathrm{oncat}\left[\alpha_{d}⊙D_{senet},\alpha_{d}⊙D_{gin}\right],\alpha_{P}=\alpha_{p}⊙P_{senet} \end{array}$$

where ⊙ is an element-wise product. The drug–target interaction weight $I_{dp}$ can be interpreted as modeling the significant semantic correlations between target proteins and compound features.

(14) $$I_{dp}=gap\left(Concat\left[gmp\left(\alpha_{D}\right),gmp\left(\alpha_{P}\right)\right]\right)$$

where gap is the global average pooling operation.

### 2.5. Drug and Target Protein Binding Affinity Prediction

Finally, interaction information $I_{dp}$ was fed directly into MLPs to map the drug–target affinity score. Here, this MLPs consists of four layers, each followed by a ReLU and dropout layer, which are applied to alleviate the model from over-fitting.

(15) $$\begin{array}{c} DTA=MLP(I_{dp}). \end{array}$$

## 3. Materials and Methods

### 3.1. Benchmark Datasets

This research assessed the DoubleSG-DTA with three benchmark datasets: Davis [32], KIBA [33], and BindingDB [34] datasets. The statistics of the Davis, KIBA, and BindingDB datasets and split strategy have been listed in Table 1.

(16) $$\begin{array}{c} pK_{d}=-\log_{10}\frac{K_{d}}{1\times 10^{9}} \end{array}$$

The Davis dataset was highly biased and discrete. We converted the $K_{d}$ values into log space according to Equation (16) [8], and the KIBA dataset comprises KIBA scores for about 118 K protein–compound interactions, and KIBA scores were derived from different bioactivity measures, such as $K_{i}$, $K_{d}$, or $IC_{50}$. The BindingDB dataset collects binding affinities for small molecule drugs and target proteins for public access.

### 3.2. Evaluation Metrics

To ensure consistency and a fair comparison, we applied the Concordance index (CI, ↑), Mean Square Error (MSE, ↓), and Regression toward the mean ($r_{m}^{2}$ index, ↑) as performance metrics following previous studies [8,11,13] to assess the model.

MSE: The MSE metric was commonly used to measure the difference between the ground truths and the predicted values, and minimizing the MSE was the main training objective.

CI: The CI metric was introduced to measure the probability of the concordance between the ground truths and the predicted values. CI values range between 0.50 and 1.0, with values less than 0.7 indicating less convincing model prediction, 0.71 to 0.90 indicating moderate prediction accuracy, and more than 0.9 indicating reliable predictions.

$r_{m}^{2}$: The $r_{m}^{2}$ metric was extensively adopted to evaluate the external predictive performance of regression-based models, and an acceptable model has a $r_{m}^{2}$ value greater than 0.5.

(17) $$\begin{array}{c} MSE=\frac{1}{N}\sum_{i=1}^{N}{\left(DTA{}_{i}-Label{}_{i}\right)}^{2} \end{array}$$

$DTA{}_{i}$ and $Label{}_{i}$ mean the predictive value and the ground truth, respectively.

(18) $$\begin{array}{c} CI=\frac{1}{Z}\sum_{\delta_{i}>\delta_{j}}^{}\zeta \left(DTA{}_{max}-DTA{}_{min}\right) \end{array}$$

$DTA{}_{max}$ and $DTA{}_{min}$ represent the predictive values of the highest affinity $\delta_{i}$ and the lowest affinity $\delta_{j}$. ζ(x) expresses the step function [15], where $\zeta \left(x\right)=\left\{1,x>0;0.5,x=0;0,x<0;\right\}$, *Z* is a normalization constant.

(19) $$\begin{array}{c} r_{m}^{2}=r^{2}\times \left(1-\sqrt{r^{2}-r_{0}^{2}}\right). \end{array}$$

Generally, an acceptable model has a $r_{m}^{2}$ value greater than 0.5, where the $r_{0}^{2}$ and $r^{2}$ designate squared correlation coefficients of interception or not.

More importantly, the Pearson correlation coefficient was employed to measure the linear correlation between the ground truths and predicted values. The Pearson correlation coefficient can be calculated as follows.

(20) $$\begin{array}{c} Pearson\left(DTA,Label\right)=\frac{Cov\left(DTA,Label\right)}{\sigma \left(DTA\right)\sigma \left(Label\right)}, \end{array}$$

where Cov means co-variance, and σ represents the standard deviation.

### 3.3. Hyperparameter Settings

Experiments were conducted with an NVIDIA RTX A5000 GPU. We adopted five-fold cross-validation to evaluate the quality of previously reported works and DoubleSG-DTA model, Table 2 gives the hyperparameter settings in experiments.

### 3.4. Baselines

In this part, we conducted experiments applying the MSE(↓), CI(↑), and $r_{m}^{2}$(↑) to assess the DoubleSG-DTA method and previous studies on the above three benchmark datasets, including DeepDTA [8], GraphDTA [11], MATT-DTI [13], AttentionDTA [16], DeepCDA [17], and DMIL-PPDTA [18]. Besides, we also benchmarked our work against proteochemometrics methods [35], including the support vector machine (SVM), feedforward neural network (FNN), SimBoost [12], Random Forest (RF) [14], and KronRLS [15].

## 4. Results and Discussion

### 4.1. Comparison against Baselines in Regression Tasks

Table 3, Table 4 and Table 5 summarize the quantitative results of the DoubeSG-DTA and previously studied models on the benchmark datasets. Obviously, DoubleSG-DTA achieved significantly superior performances to other regression-based methods on various datasets.

Considering the Davis dataset, the MSE metric of the DoubleSG-DTA model was 0.219, 0.004 lower than the best DMIL-PPDTA [18] model in the sequence-based models, and the CI and $r_{m}^{2}$ metrics of our model were 0.902 and 0.725, 0.009 and 0.04 higher than FNN [20] model in the sequence-based models, respectively. When comparing with the best GraphDTA [11] model in the graph-based models, the CI value was increased by 0.009 and the MSE value was decreased by 4.37%.

Considering the KIBA dataset, the MSE and $r_{m}^{2}$ metric of the DoubleSG-DTA model were 0.138 and 0.787, 6.12% lower and 0.003 higher than the best DMIL-PPDTA [18] model in the sequence-based models, and the CI metrics of our model were 0.896, 0.007 higher than the MATT-DTI [13] model in the sequence-based models, respectively. When compared with the best GraphDTA [11] model in the graph-based models, the CI value was increased by 0.005 and the MSE value was decreased by 0.001.

Considering the BindingDB dataset, the MSE metric of the DoubleSG-DTA model was 0.533, 11.61% lower than the best AttentionDTA [16] model in the sequence-based models, and the CI and $r_{m}^{2}$ metrics were 0.862 and 0.726, which were 0.01 and 0.039 higher than it, respectively. When compared with the best GraphDTA [11] model in the graph-based models, the CI and $r_{m}^{2}$ metrics were increased by 0.005 and 0.023, respectively, and the MSE metric was decreased by 4.31%.

**Figure 2:**
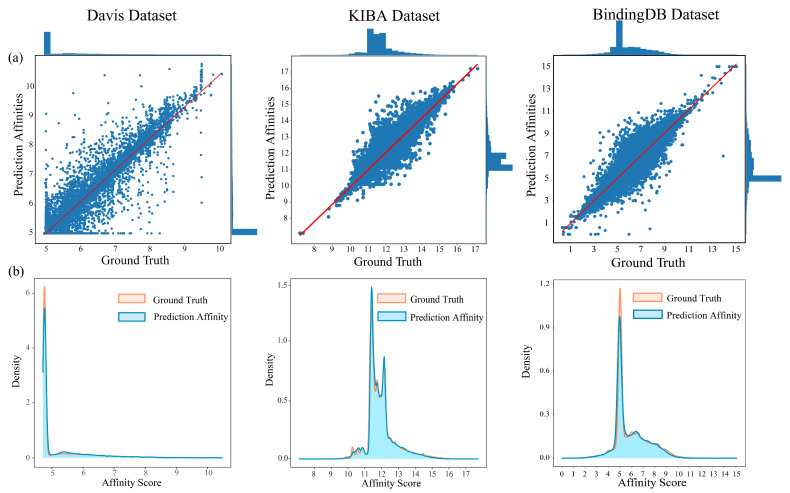
Correlation distribution between ground truths and predictive values on benchmark datasets, (**a**) scatter and (**b**) kernel density estimate plots.

Figure 2 presents that the predictive values and ground truths show approximately overlapping distribution trends in the KIBA, Davis, and BindingDB datasets. In addition, using the Pearson correlation enabled us to make an unbiased assessment for DoubleSG-DTA that is optimized for MSE. In particular, our model achieved even better Pearson correlations of 0.852, 0.894, and 0.867 in the three benchmark datasets, respectively.

These results indicate that the powerful graph isomorphism networks, coupled with the lightweight squeeze-and-excitation networks enable the DoubleSG-DTA to perform exceptionally well under the support of cross-multi-head attention.

### 4.2. Ablation Study 1: The Effect of Graph Isomorphism Network Layers on Model Performance

**Figure 3:**
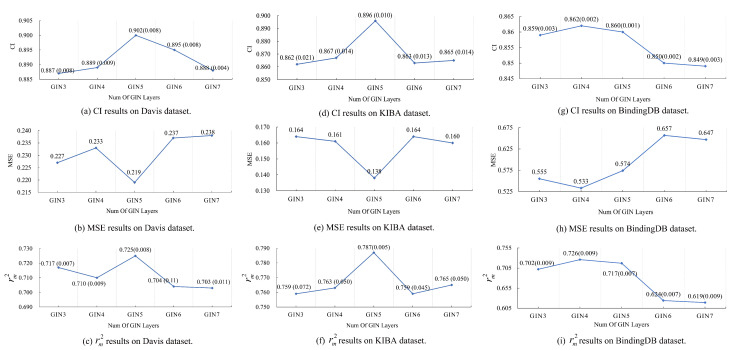
Impact of the layers of the graph isomorphism network on the performance of DoubleSG-DTA.

Extracting drug representations highly relies on the graph computational capability of GIN. We conducted an ablation experiment to investigate the contribution of graph isomorphism network depth on prediction performance. It can be seen from Figure 3 that the DoubleSG-DTA outperforms all other settings when the count of layers of GINs $L\in \left\{4,5\right\}$, and the CI and $r_{m}^{2}$ metrics of the DoubleSG-DTA model tend to decrease as the number of GIN layers increases, and the MSE metric of the main objective of DoubleSG-DTA training increases sharply. GIN performs a weighted average of its own features and near and far neighboring node features to update the node’s new features, with the aim of capturing graph representations and discriminating between graph structures. However, increasing the number of layers infinitely will cause the feature vectors of nodes within the same cluster to gradually converge to similarity, which may lead to node-wise over-smoothing and impair model decision-making performance [36]. Therefore, the appropriate depth of GIN facilitates obtaining drug graph representations, while stacking a collection of GIN layers may cause over-smoothing and vanishing gradients problems.

### 4.3. Ablation Study 2: The Effect of Se Block on Model Performance

This work forgoes the CNNs used in previous studies [8,13,16,17] as the feature extractor but instead creates multilayer squeeze-and-excitation networks to construct textual features of drug and amino acid sequences, which was compared with a CNN-based method. As shown in Table 6, although the multilayer SE modules with channel attention were embedded into the DoubleSG-DTA model that caused the model parameters to rise and also caused higher model complexity, there was no significant increase in the training time of the model on the three benchmark datasets. Therefore, controlled experiments demonstrated that the DoubleSG-DTA model with SENet blocks (DoubleSG-DTA + SENet) achieves considerable improvements at a slightly additional computational burden than the models without it (DoubleSG-DTA + CNN). Overall, our findings suggest that SENets significantly reduce the model’s error rate, which benefits from inter-channel attention.

### 4.4. Ablation Study 3: Interaction Learning with Cross-Multi-Head Attention Mechanism

Ultimately, this study investigated the impact of the cross-multi-head attention mechanism modeling the reality-based molecular docking behavior of drug molecules and target proteins, and compared it against the method of concatenating both. As shown in Table 7, the MSE index of the DoubleSG-DTA model with cross-multi-head attention decreased by 9.50%, 10.39%, and 3.79% compared to the latter in the Davis, KIBA, and BindingDB datasets, respectively. Besides, the $r_{m}^{2}$ index increased by 0.012, 0.014, and 0.024. Overall, after using the cross-multi-head attention mechanism, the complete DoubleSG-DTA model led to more considerable improvements.

## 5. Case Study on the NSCLC with ${\mathrm{EGFR}}^{T790M}$ Mutation

According to the statistics of cancer data in 2021 [37], lung cancer mortality increased to around 46% of total cancer mortality, among which NSCLC accounted for approximately 85% of lung malignancies. Patients with NSCLC are normally accompanied by epidermal growth factor receptor (EGFR) mutations [38], which brings great challenges to the treatment of NSCLC. In recent years, the remarkable achievements of small-molecule EGFR tyrosine kinase inhibitors (EGFR-TKIs) in targeted therapy have brought light to NSCLC patients. First-generation EGFR-TKIs (Gefitinib and Erlotinib) and second-generation EGFR-TKI (Afatinib) significantly improved the prognosis of advanced NSCLC patients compared to platinum-based chemotherapy. Unfortunately, the majority of patients develop $EGFR^{T790M}$ mutation, resulting in severe resistance symptoms [39]. Inevitably, despite the high selectivity of the third-generation EGFR-TKI (Osimertinib) targeting NSCLC harboring $EGFR^{T790M}$ mutation, patients develop secondary resistance [40].

Natural products continue to be a precious source of templates with structural complexity and numerous pharmacophores in drug R&D, especially effective in cancer. For instance, paclitaxel [41] and vincristine [42] have been widely invested in the clinical treatment of tumors. In this section, we preferred to screen high-affinity and good properties targeted inhibitors of NSCLC with $EGFR^{T790M}$ mutation from natural products. We hope our results may provide clues for medical scientists to develop highly selective natural drugs.

For the above purpose, we acquired the FASTA sequence of mutant protein $EGFR^{T790M}$ (PDB ID:2JIT [28]) from the Protein Data Bank [43] and collected 2645 natural compounds from Selleck Chemicals https://www.selleck.cn/ (accessed on 4 January 2023), which are easily optimized for good human oral bioavailability (OB > 40%) and drug-likeness (DL > 0.18) [44,45]. Table 8 provides information on the top 10 natural products predicted by DoubleSG-DTA, which have the highest affinity to the $EGFR^{T790M}$ mutant protein.

Then, we carried out a comprehensive literature survey on the top 10 natural products. Based on the study [46], gossypol not only significantly increased the sensitivity to EGFR-TKIs in H1975 cells carrying $EGFR^{L858R/T790M}$, but inhibited cell proliferation and induced apoptosis. The Gö6976 is derived from Staurosporine, experimental confirmation that Gö6976 (at 500 nanomolar) exhibits significant binding affinity for $EGFR^{T790M}$ mutants, while it shows a significantly lower affinity for wild-type EGFR [47]. The research results indicate that Shikonin has selective cytotoxic effects on gefitinib-resistant NSCLC cell lines carrying ${EGFR}^{T790M}$ mutation, while relatively safe to normal lung cells [48]. Gossypol acetic acid significantly enhances sensitized lung cancer cells carrying ${EGFR}^{L858R/T790M}$ mutation to gefitinib and overcomes EGFR-TKIs resistance [49,50]. According to the above-mentioned report, such natural products may be promising strategies to combat resistance in NSCLC harboring ${EGFR}^{T790M}$ mutation.

**Table 8:** Docking information of the top 10 natural products with the highest affinity.

Natural Products	MF	MW	H-Bonds	Binding-Energy (KJ/mol)
Gossypol [46]	C_30_H_30_O_8_	518.60	4	−12.636
Gossypol acetic acid [50]	C_32_H_34_O_10_	578.60	3	−14.644
Staurosporine [47]	C_28_H_26_N_4_O_3_	466.50	3	−18.744
Emodin	C_15_H_10_O_5_	270.24	4	−13.933
Physcion	C_16_H_12_O_5_	284.26	3	−16.862
Aurantio-obtusin	C_17_H_14_O_7_	330.29	4	−17.531
Shikonin [48]	C_16_H_16_O_5_	288.29	3	−13.180
Rhein	C_15_H_8_O_6_	284.22	6	−16.192
Obtusifolin	C_16_H_12_O_5_	284.26	3	−15.104
Chrysophanol	C_15_H_10_O_4_	254.24	5	−16.025

## 6. Molecular Docking and Biological Interpretation

**Figure 4:**
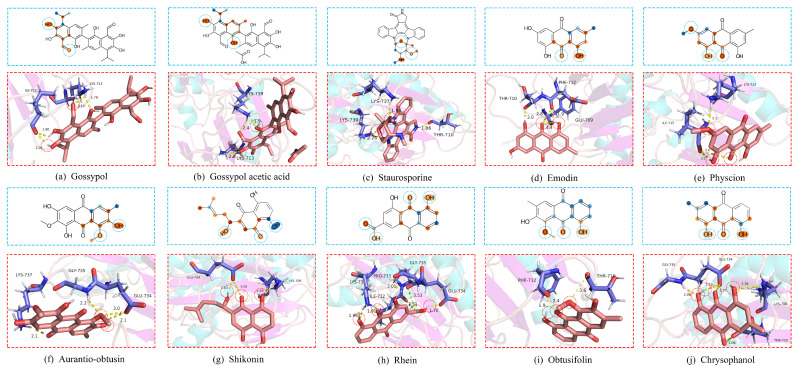
The blue box shows the heatmaps of atomic contributions. In the red box, are molecular docking poses of the top 10 natural drugs with $EGFR^{T790M}$ mutant proteins.

To further validate such new interactions, computational docking was performed via AutoDock [51]. As shown in Figure 4, we employed the most efficient, reliable, and successful Lamarckian genetic algorithm in Autodock to perform an adaptive global–local search for the lowest-energy ligand–receptor docked conformation, and predicted the binding free energy via an empirical binding free energy force field [52]. The ligand–receptor binding energy includes electrostatic interactions, hydrogen bonding, van der Waals forces and hydrophobic interactions, and so forth, and the structural stability is negatively correlated with the binding energy value. Furthermore, an acceptable molecular docking conformation that has a binding energy of less than −5.0208 KJ/mol. Drug molecule ligands interact stably with target proteins in the above manner, aiming to exert a variety of biological activities such as anti-inflammatory and anti-tumor activities of the drug molecules, and to stimulate the physiological and pharmacological functions of the protein. As shown in Figure 4 and Table 8, the docking indicates that the top 10 natural compounds can be stably docked to the $EGFR^{T790M}$ protein by generating multiple hydrogen bonds.

Graph neural networks have always been criticized because of their poor interpretability, and these models are commonly thought of as “black boxes”. In this work, inspired by Grad-AAM [20] and Grad-CAM [53], which employed the gradient-weighted class activation mapping method, the regions of graph structure that contribute most to the prediction results are visualized as heatmaps, enhancing the interpretability of deep learning-based network models processing graph data.

Since the last layer of the GINs of DoubleSG-DTA incorporates the richest high-level semantic information, the drug graph representations are visualized to produce heatmaps depicting the atoms and functional groups that contribute most prominently to predicting DTA. We denote the feature map of the last graph convolution layer as *F*. In order to obtain the probability map *P* of atomic node *v* for a given drug molecule, we calculate the gradient of the predicted affinity DTA of the molecule binding to the target protein at the *c*-th channel of the feature map *F* and atomic node *v*. The gradient $W_{c}$ has been calculated as follows.

(21) $$\begin{array}{c} W_{c}=\frac{1}{\left|V\right|}\sum_{v\in V}\frac{\partial DTA}{\partial F_{v}^{c}}. \end{array}$$

Next, a weighted combination of the data for each channel of the feature map F was performed, followed by the ReLU activation function.

(22) $$\begin{array}{c} P=\delta \left(\sum_{c}W_{c}F_{}^{c}\right). \end{array}$$

Finally, the gradient weights were scaled to the range of 0 to 1 using min–max normalization to obtain a probability map *P* of the weighted distribution of the drug molecules, which was further rendered into a heatmap.

As shown in Figure 4, the active structures in the heatmaps overlap with molecular docking sites by more than 77.14%, and the mathematical calculation formulation is given as Equation (23). Figure 4 explains that describing the drug molecules as graphs and learning the topological pattern structures of the drug molecules with an appropriate depth of GIN can accurately discriminate between drug molecular active structures.

(23) $$\begin{array}{c} overlap\;rate=\frac{1}{N}\sum_{i=1}^{N}\left(\frac{P_{drug}}{P_{protein}}\right), \end{array}$$

where *N* denotes the number of drugs, $P_{protein}$ stands for the number of molecular docking sites, and $P_{drug}$ is the number of atoms and functional groups that contributes the most and is identical to the molecular docking site.

## 7. Conclusions

This investigation presented an interpretable deep learning-based computational model to project the affinity of drug–target pairs for aiding in drug discovery. The experimental results indicated that the simple yet powerful graph isomorphism networks coupled with the lightweight squeeze-and-excitation networks made the DoubleSG-DTA perform exceptionally well with the support of cross-multi-head attention compared with all previously reported works. Extensive experiments have revealed that (i) the most appropriate number of graph isomorphism network layers for extracting drug graph representations and discriminating between molecular structures is $\left\{4,5\right\}$, (ii) the SE block with the soft attention mechanism selectively emphasized information features by expanding the perceptual field, significantly boosting the model’s decision making, and (iii) fully modeling the interaction between compounds and proteins facilitates further performance in predicting drug–target binding affinity. Ultimately, the well-established DoubleSG-DTA was applied to screen promising high-affinity compounds of Non-Small Cell Lung Cancer with $EGFR^{T790M}$ mutation from natural products to provide some clues for medical scientists. In addition, drug graph representations were visualized as heatmaps, in which the active structures that contributed the most covered almost all molecular docking sites, which may provide biological interpretation and entry points for later molecular optimization. Overall, DoubleSG-DTA may be an effective in silico drug discovery tool for medical challenges and urgent public health emergencies.

## Figures and Tables

**Table 1:** The detailed statistics of Davis, KIBA, and BindingDB datasets.

Dataset	No. Proteins	No. Drugs	No. Interactions	Interactions		
				Train Data	Validation Data	Test Data
Davis	442	68	30,056	20,037	5009	5010
KIBA	229	2111	118,254	78,836	19,709	19,709
BindingDB	1620	18,044	56,525	37,684	9421	9420

**Table 2:** The hyperparameters of DoubleSG-DTA.

Hyperparameters	Davis Dataset	KIBA Dataset	BindingDB Dataset
Embedding Size	128	128	128
SENet layers	3	3	3
GIN layers	[3, 4, 5, 6, 7]	[3, 4, 5, 6, 7]	[3, 4, 5, 6, 7]
Number of filters in SENets	[16, 32, 48]	[32, 64, 96]	[32, 64, 96]
Hidden size in MLPs	[1024, 1024, 512]	[1024, 1024, 512]	[1024, 1024, 512]
Number of attention heads	8	8	8
Epoch	600	600	600
Learning rate	0.0001	0.0001	0.0001
Batch Size	512	1024	1024
Dropout rate	0.2	0.2	0.2
Optimizer	Adam	Adam	Adam
Activation Function	ReLU	ReLU	ReLU
Loss Function	MSEloss	MSEloss	MSEloss

**Table 3:** Comparison of previous studies and the DoubleSG-DTA on the Davis dataset.

Dataset	Methods	Protein	Compounds	Interaction	CI(std)↑	MSE↓	$r_{m}^{2}$(std)↑
Davis	Random Forest [14]	ECFP	PSC	—	0.854 (0.002)	0.359	0.549 (0.005)
	SVM [20]	ECFP	PSC	—	0.857 (0.001)	0.383	0.513 (0.003)
	FNN [20]	ECFP	PSC	—	0.893 (0.003)	0.244	0.685 (0.015)
	KronRLS [15]	Smith-Waterman	Pubchem Sim	—	0.871 (0.001)	0.379	0.407 (0.005)
	SimBoost [12]	Smith-Waterman	Pubchem Sim	—	0.872 (0.001)	0.282	0.644 (0.006)
	DeepDTA [8]	CNN	CNN	Concatention&FCN	0.878 (0.004)	0.261	0.630 (0.017)
	DeepCDA [17]	CNN&LSTM ^1^	CNN&LSTM	Two-sided Attention&FCN	0.891 (0.003)	0.248	0.649 (0.009)
	MATT-DTI [13]	CNN	CNN&Relation-aware Self-Attention	Multi-head Attention&FCN	0.891 (0.002)	0.227	0.683 (0.017)
	AttentionDTA [16]	CNN	CNN	Multi-head Attention&FCN	0.887 (0.005)	0.245	0.657 (0.024)
	DMIL-PPDTA [18]	Transformer	Transformer	Multi-head attention&FCN	0.880 (0.007)	0.223	0.642 (0.017)
	GraphDTA [11]	CNN	GIN	Concatention&FCN	0.893 (—)	0.229	—
	GraphDTA [11]	CNN	GAT	Concatention&FCN	0.892 (—)	0.232	—
	GraphDTA [11]	CNN	GCN	Concatention&FCN	0.890 (—)	0.254	—
	GraphDTA [11]	CNN	GAT&GCN	Concatention&FCN	0.881 (—)	0.245	—
	DoubleSG-DTA	CNN	GIN+CNN ^2^	Concatention&FCN	0.886 (0.003)	0.250	0.688 (0.031)
	**DoubleSG-DTA**	**SENet**	**GIN+SENet**	**Cross-Multi-head Attention&FCN**	**0.902 (0.008)**	**0.219**	**0.725 (0.008)**

^1^ & stands for concatenating learning. ^2^ + stands for parallel learning. Bold text indicates the best result.

**Table 4:** Comparison of previous studies and the DoubleSG-DTA on the KIBA dataset.

Dataset	Methods	Protein	Compounds	Interaction	CI(std)↑	MSE↓	$r_{m}^{2}$(std)↑
KIBA	Random Forest [14]	ECFP	PSC	—	0.837 (0.000)	0.245	0.581 (0.000)
	SVM [20]	ECFP	PSC	—	0.799 (0.001)	0.308	0.513 (0.004)
	FNN [20]	ECFP	PSC	—	0.818 (0.005)	0.216	0.659 (0.015)
	KronRLS [15]	Smith-Waterman	Pubchem Sim	—	0.782 (0.001)	0.411	0.342 (0.001)
	SimBoost [12]	Smith-Waterman	Pubchem Sim	—	0.836 (0.001)	0.222	0.629 (0.007)
	DeepDTA [8]	CNN	CNN	Concatention&FCN	0.863 (0.002)	0.194	0.673 (0.009)
	DeepCDA [17]	CNN&LSTM	CNN&LSTM	Two-sided Attention&FCN	0.889 (0.002)	0.176	0.682 (0.008)
	MATT-DTI [13]	CNN	CNN&Relation-aware Self-Attention	Multi-head Attention&FCN	0.889 (0.001)	0.150	0.756 (0.011)
	AttentionDTA [16]	CNN	CNN	Multi-head Attention&FCN	0.882 (0.004)	0.162	0.735 (0.003)
	DMIL-PPDTA [18]	Transformer	Transformer	Multi-head attention&FCN	0.881 (0.003)	0.147	0.784 (0.006)
	GraphDTA [11]	CNN	GIN	Concatention&FCN	0.882 (—)	0.147	—
	GraphDTA [11]	CNN	GAT	Concatention&FCN	0.866 (—)	0.179	—
	GraphDTA [11]	CNN	GCN	Concatention&FCN	0.889 (—)	0.139	—
	GraphDTA [11]	CNN	GAT&GCN	Concatention&FCN	0.891 (—)	0.139	—
	DoubleSG-DTA	CNN	GIN+CNN	Concatention&FCN	0.856 (0.002)	0.164	0.721 (0.009)
	**DoubleSG-DTA**	**SENet**	**GIN+SENet**	**Cross-Multi-head Attention&FCN**	**0.896 (0.010)**	**0.138**	**0.787 (0.005)**

Bold text indicates the best result.

**Table 5:** Comparison of previous studies and the DoubleSG-DTA on the BindingDB dataset.

Dataset	Methods	Protein	Compounds	Interaction	CI(std)↑	MSE↓	$r_{m}^{2}$(std)↑
BindingDB	KronRLS [15]	Smith-Waterman	Pubchem Sim	—	0.815 (0.003)	0.939	—
	DeepDTA [8]	CNN	CNN	Concatention & FCN	0.826 (0.001)	0.703	0.669 (0.004)
	DeepCDA [17]	CNN & LSTM	CNN & LSTM	Two-sided Attention & FCN	0.822 (0.001)	0.844	0.631 (0.002)
	AttentionDTA [16]	CNN	CNN	Multi-head Attention & FCN	0.852 (0.003)	0.603	0.687 (0.013)
	GraphDTA [11]	CNN	GIN	Concatention & FCN	0.857 (—)	0.557	0.703 (—)
	GraphDTA [11]	CNN	GAT	Concatention & FCN	0.817 (—)	0.929	0.555 (—)
	GraphDTA [11]	CNN	GCN	Concatention & FCN	0.850 (—)	0.638	0.647 (—)
	GraphDTA [11]	CNN	GAT & GCN	Concatention & FCN	0.855 (—)	0.593	0.682 (—)
	DoubleSG-DTA	CNN	GIN+CNN	Concatention & FCN	0.853 (0.001)	0.624	0.642 (0.008)
	**DoubleSG-DTA**	**SENet**	**GIN+SENet**	**Cross-Multi-head Attention&FCN**	**0.862 (0.002)**	**0.533**	**0.726 (0.009)**

Bold text indicates the best result.

**Table 6:** Investigating the contributions of SENet on Davis, KIBA, and BindingDB datasets.

Dataset	Methods	Protein	Compounds	Interaction	CI(std)↑	MSE↓	$r_{m}^{2}$(std)↑	Time ^1^ (std)
Davis	DoubleSG-DTA	CNN	GIN+CNN	Cross-Multi-head Attention&FCN	0.897 (0.008)	0.229	0.713 (0.077)	4.102 (0.061)
	**DoubleSG-DTA**	**SENet**	**GIN+SENet**	**Cross-Multi-head Attention&FCN**	**0.902 (0.008)**	**0.219**	**0.725 (0.008)**	4.139 (0.066)
KIBA	DoubleSG-DTA	CNN	GIN+CNN	Cross-Multi-head Attention&FCN	0.887 (0.014)	0.147	0.760 (0.048)	19.619 (0.357)
	**DoubleSG-DTA**	**SENet**	**GIN+SENet**	**Cross-Multi-head Attention&FCN**	**0.896 (0.010)**	**0.138**	**0.787 (0.005)**	20.023 (0.109)
BindingDB	DoubleSG-DTA	CNN	GIN+CNN	Cross-Multi-head Attention&FCN	0.854 (0.001)	0.614	0.646 (0.009)	13.787 (0.203)
	**DoubleSG-DTA**	**SENet**	**GIN+SENet**	**Cross-Multi-head Attention&FCN**	**0.862 (0.002)**	**0.533**	**0.726 (0.009)**	14.276 (0.165)

^1^**Time** (s) denotes the time that our proposed DoubleSG-DTA model took to train an epoch.

**Table 7:** Investigating the contributions of the cross-multi-head attention mechanism on Davis, KIBA, and BindingDB datasets.

Dataset	Methods	Protein	Compounds	Interaction	CI(std)↑	MSE↓	$r_{m}^{2}$(std)↑	Pearson↑
Davis	DoubleSG-DTA	SENet	GIN+SENet	Concatenation&FCN	0.892 (0.007)	0.242	0.713 (0.026)	0.845
	**DoubleSG-DTA**	**SENet**	**GIN+SENet**	**Cross-Multi-head Attention&FCN**	**0.902 (0.008)**	**0.219**	**0.725 (0.008)**	**0.852**
KIBA	DoubleSG-DTA	SENet	GIN+SENet	Concatenation&FCN	0.878 (0.018)	0.154	0.773 (0.063)	0.880
	**DoubleSG-DTA**	**SENet**	**GIN+SENet**	**Cross-Multi-head Attention&FCN**	**0.896 (0.010)**	**0.138**	**0.787 (0.005)**	**0.894**
BindingDB	DoubleSG-DTA	SENet	GIN+SENet	Concatenation&FCN	0.859 (0.002)	0.554	0.702 (0.009)	0.862
	**DoubleSG-DTA**	**SENet**	**GIN+SENet**	**Cross-Multi-head Attention&FCN**	**0.862 (0.002)**	**0.533**	**0.726 (0.009)**	**0.867**

## Data Availability

The source code at https://github.com/YongtaoQian/DoubleSG-DTA (accessed on 4 January 2023).

## Abbreviations

The following abbreviations are used in this manuscript:

DoubleSG-DTA	Double Sequence and Graph to Predict drug–target Affinity
DTA	drug–target affinities
EGFR	epidermal growth factor receptor
EGFR-TKIs	EGFR tyrosine kinase inhibitors
NSCLC	Non-Small Cell Lung Cancer
T790M	threonine 790 mutations
R& D	Research and Development
SMILES	Simplified Molecular Input Line Entry System
GIN	Graph Isomorphism Network
SENet	Squeeze-and-Excitation Network
MLP	Multilayer Perceptrons
ReLU	Rectified Linear Unit activation function
gap	global average pooling
gmp	global max pooling
RF	Random Forest
SVM	Support Vector Machine
FNN	Feedforward Neural Network
CI	Concordance index
MSE	Mean Square Error
$r_{m}^{2}$	Regression toward the mean
MF	Molecular Formula
MW	Molecular Weight(g/mol)
H-Bonds	Hydrogen Bonds
